# Development and validation of a Q-PCR based TCID_50_ method for human herpesvirus 6

**DOI:** 10.1186/1743-422X-9-311

**Published:** 2012-12-18

**Authors:** Rasmus K L Gustafsson, Elin E Engdahl, Anna Fogdell-Hahn

**Affiliations:** 1 Karolinska Institutet, Department of Clinical Neuroscience, The Multiple Sclerosis Research Group, Center for Molecular Medicine building L8:00, Karolinska University hospital Solna, SE-171 76, Stockholm, Sweden

**Keywords:** HHV-6A, Viral titer, TCID50, Q-PCR, Immunofluorescence assay, Ocular inspection

## Abstract

**Background:**

For titer assessment of human herpesvirus 6 (HHV-6), IFA targeting viral proteins or a TCID_50_ method with ocular inspection for CPE can be used. These methods rely on the subjective decision of the assessor, obstructing the ability to obtain unanimous results.

**Findings:**

We have developed and validated an alternative TCID_50_ read-out approach where infection in the titration culture plate is assessed by viral DNA load change by quantitative PCR. A ten time increase in viral DNA load was determined as cut point for infection since that yielded a maximum correlation with viral protein expression (93%). The average intra-assay CV was 9% for quantitative PCR read-out of TCID_50_ compared to 45% for ocular inspection read-out of TCID_50_, 14% for IFA read-out of TCID_50_, and 43% for an infectious units approach using IFA. The average inter-assay CV for quantitative PCR read-out of TCID_50_ was 73%, compared to 66%, 25% and 77% for the ocular inspection read-out for TCID_50_, IFA read-out of TCID_50_ and infectious unit approaches respectively.

**Conclusions:**

The quantitative PCR based read-out of TCID_50_ proved to be more robust and easier to interpret than traditional TCID_50_ assessment approaches for HHV-6, and therefore it might be considered as an alternative method.

## Findings

It is crucial to have control of the viral titer in experimental work with viruses. To facilitate comparisons between studies performed at different laboratories the use of harmonized standard methods are desirable. For human herpesvirus 6 (HHV-6) [1], a β-herpesvirus that most people have been exposed to [2,3], the 50% tissue culture infectivity dose (TCID_50_) method [4] is often used for viral titer assessment. A commonly used read-out is ocular inspection for cytopathic effects (CPE), i.e. enlargement of the infected cells [5]. One obstacle with this approach is that cells may enlarge even when not infected. It is especially difficult at the borderline of infection in titration series’ as cells enlarged due to infection tend to enlarge less with increased dilution of the virus (Additional file 1: Figure S1). Immunofluorescence assay (IFA) is an alternative read-out approach to ocular inspection in TCID_50_ assessment [6] or for calculation of infectious units, i.e. the fraction of infected cells [7]. The IFA based read-out is more distinct in discriminating infected from uninfected cells but the staining is laborious and a substantial number of cells need to be counted to get reliable values. The monitoring of individual cells implies a risk to misinterpret a cell’s positivity, a disadvantage of both ocular inspection and IFA based read-outs. Hence, we developed and validated an alternative read-out approach of TCID_50_ where the increase in viral DNA load is measured in every titration well of TCID_50_ culture plates using real-time quantitative PCR (Q-PCR). This approach was compared with ocular inspection and IFA read-outs of TCID_50_, and with the infectious units approach described above.

HHV-6A (GS strain) [1] was propagated in the T-cell line HSB-2 in GlutaMAX containing RPMI 1640 medium (Invitrogen, United Kingdom) supplemented with 10% fetal bovine serum (HyClone, UT), 100 U/ml penicillin and 100 μg/ml streptomycin (Invitrogen). When approximately 50% of the live cells had enlarged, the supernatant was harvested and frozen immediately in aliquots at −80°C until analysis. As controls, virus supernatant of passage 17 (P17) were inactivated by UV light for 20 min or with heat treatment at 56°C for 1 h. HHV-6A replication in HSB-2 cells was followed for ten days using Q-PCR (Applied Biosystems, United Kingdom) as previously described [8]. Prior to Q-PCR analysis, DNA was extracted from the cell suspensions using a 96-well plate bead-based kit according to the manufacturer’s protocol (MagMAX-96 Viral RNA Isolation Kit, Applied Biosystems). To assess the HHV-6A DNA content in the viral batches, Q-PCR was performed as described above after DNA extraction using filter columns (QIAGEN GmbH, Germany).

To set up the TCID_50_ culture plates, cell suspensions of 40 μl 10^4^ HSB-2 cells per well were seeded in round bottom 96-well culture plates. The cells were inoculated for 3–4 hours with 160 μl of five-fold dilutions of HHV-6A supernatant, six replicates per dilution. Mock and medium controls were included in triplicate wells on all plates. The cells were washed once before 50–70 μl of cell suspension were sampled from every well and stored at −80°C as zero day post infection (dpi) samples. The remaining cell suspensions were incubated for seven days at 37°C. At seven dpi, the thawed zero dpi plate and the seven dpi plates were subjected to DNA extraction using the bead-based kit described above. Thereafter the viral DNA was quantified by Q-PCR as described above.

For IFA the cells were fixed onto glass slides with a 1:1 mixture of acetone and methanol at −20°C for 10 minutes, blocked with 5% goat serum and 3% BSA in PBS, and stained with a primary mouse monoclonal antibody specific to the HHV-6 glycoprotein gp116/54/64 (Advanced Biotechnologies, MD). The staining was visualized by an Alexa 633 conjugated goat anti-mouse IgG (Invitrogen). Staining with 4',6-diamidino-2-phenylindole (Vector laboratories, CA) was used to visualize the cell nuclei. Cover slips were mounted with mounting media (DAKO A/S, Denmark), and the slides were analyzed using a confocal microscope (Leica Microsystems, Germany). The fraction of infected cells was determined by manual counting. If ≥1/3 of the cells contained viral protein, ≥25 cells per well were counted and if <1/3 of the cells contained viral protein, ≥100 cells per well were counted. Wells where ≥2% of the cells stained positive were considered infected. The level of viral DNA load increase in each well was correlated to the IFA staining constructing a formula in the software Excel (Microsoft, WA). This formula asks whether the viral DNA in a certain well in the TCID_50_ plate has shifted a certain number of times which is set, and if the very same well was positive in IFA or not.

For comparisons with the Q-PCR read-out, all TCID_50_ plates were assessed by ocular inspection using a phase contrast microscope by two independent inspectors (ocular TCID_50_). Wells were considered as infected if at least one enlarged cell was found. For both read-out approaches the TCID_50_ was calculated according to the Reed and Muench formula [4].

The TCID_50_ results determined by Q-PCR (Q-PCR TCID_50_) was compared with the infectious units assessment by IFA [7] (personal communication with Louis Flamand) at three time points for P19 and one for P27. Briefly, 2.5*10^5^ HSB-2 cells were inoculated as described above with various dilutions of virus in triplicate wells for every dilution. At two dpi the cells were subjected to IFA targeting the early viral protein p41 (clone 9A5D12, Santa Cruz Biotech. Inc., CA) as described above. The viral titer, expressed as infectious units per ml was calculated by multiplying the fraction of infected cells with the total number of cells at zero dpi and dilution factors.

**Figure 1:**
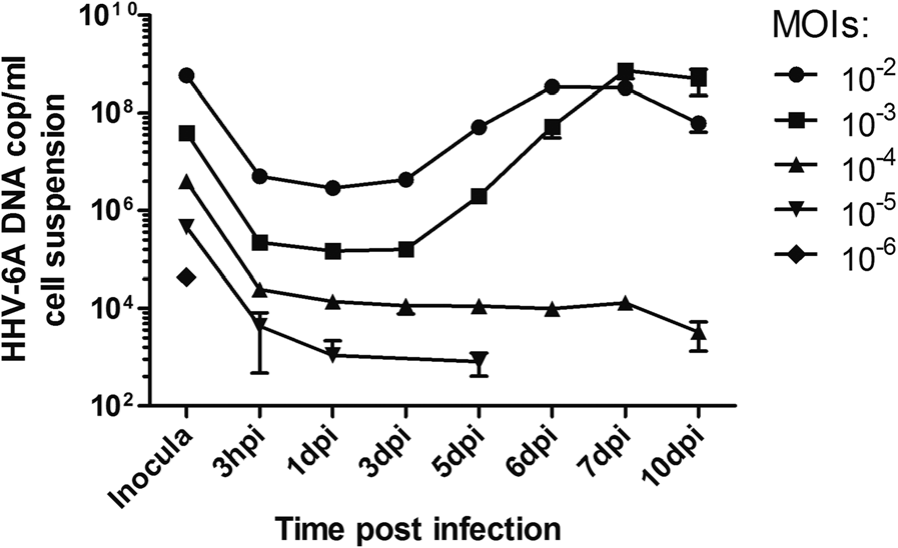
**Replication of HHV-6A (GS strain) followed by Q-PCR.** Data shown are mean results (± SEM) of triplicate cultures for every multiplicity of infection (MOI). Connecting lines discontinue when the viral DNA load dropped under the detection limit.

**Figure 2:**
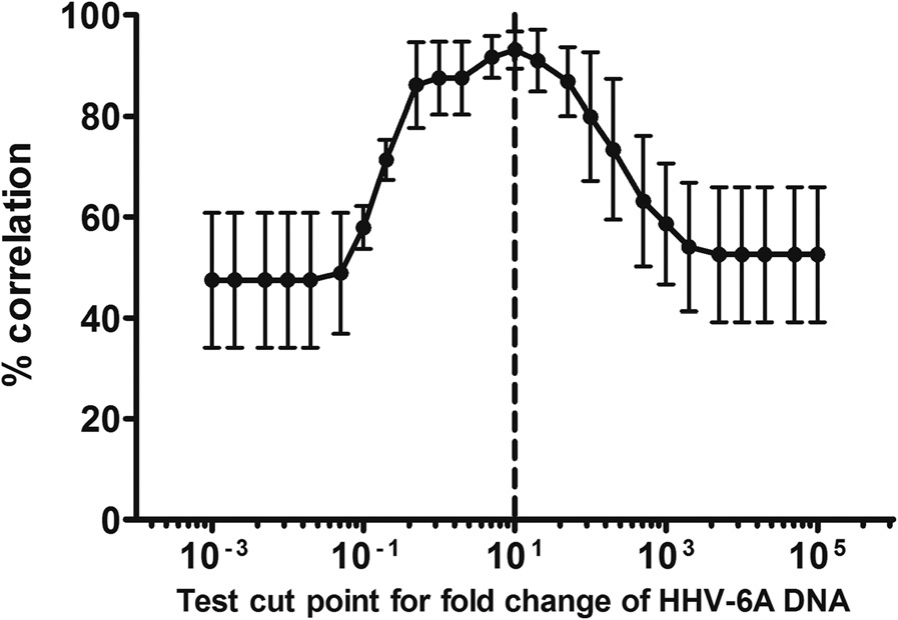
**Determination of cut-point for infection.** The optimal cut-point for infection was found to be a ten times increase where the correlation to protein expression was seen in 93% of the wells by IFA with an antibody targeting the late viral protein gp116/54/64. No well contained viral protein where the viral DNA load had not increased ten times. Data shown are mean results (± SEM) from three TCID_50_ plates.

To determine the optimal time-point for viral DNA load increase measurements, virus replication was followed for ten days. Seven days was required to reach sufficient increase in viral DNA (Figure 1) and was therefore chosen as the harvest time-point. To set the cut point of relative viral DNA increase corresponding to positive infection, the viral DNA load increase was correlated with viral protein expression. IFA was performed at seven dpi on cells from every well in three TCID_50_ plates for two different virus batches. The optimal cut point was found to be a ten time increase in viral DNA, where the correlation to protein expression was seen in 93% of the wells (Figure 2).

Comparing the values of Q-PCR TCID_50_ to ocular and IFA TCID_50_, one Q-PCR TCID_50_ equaled 1.41 ocular and 1.03 IFA TCID_50_ based on 13 or 5 assessments respectively. Q-PCR TCID_50_ did not give statistically different values compared to ocular or IFA TCID_50_ (p = 0.41 or p = 0.29 respectively) (paired t-test) (Table 1).

**Table 1:** Titer assessment for HHV-6A GS strain batches expressed as TCID_50_ determined by Q-PCR, ocular inspection or immunofluorescence assay (IFA), and as infectious units (Inf U) determined by IFA

**Batch**	**Q-PCR**	**Ocular inspection**	**Inf U/ml**	**IFA**	**HHV-6A**
**(passage)**	**TCID_50_/ml**	**TCID_50_/ml**		**TCID_50_/ml**	**DNA cop/ml**
P17	1215 ±566 (x3)^1^	771 ±466 (x3)	nd^2^	972 ±719 (x2)	7.6e8 ±1.4e8 (x2)
P19	806 ±679 (x5)	465 ±408 (x5)	2.5e4 ±1.6e4 (x3)	649 N/A^3^ (x1)	16.0e8 ±1.3e8 (x2)
P21	6 ±8 (x2)	14 ±6 (x2)	nd	nd	7.7e8 ±0.15e8 (x2)
P27	7 ±6 (x3)	30 ±15 (x3)	3.9e4 N/A (x1)	13 ±3 (3x)	nd
P17 UV-inact.	0 N/A (x1)	0 N/A (x1)	nd	nd	nd
P17 heat-inact.	20 N/A (x1)	4 N/A (x1)	nd	nd	nd

^1^ Number of replications of titer assessments for each passage.

^2^ nd: Not done.

^3^ N/A: Not applicable.

Ocular inspection was performed by two independent assessors. Data presented are means ± standard deviations.

Viral DNA copy numbers are often used as a rough estimate of the amount of viral particles a particular viral batch contains. However, it is uncertain how well this corresponds to infectivity. To assess this, TCID_50_ values were compared with the viral DNA copy numbers for the respective batch. The average ratios of viral DNA load to Q-PCR TCID_50_ values in the virus batches were similar for P17 and P19; 6.3*10^5^ and 2.0*10^6^ viral DNA copies per TCID_50_ respectively. For P21 however, the ratio was considerably higher, 1.3*10^8^ viral DNA copies per TCID_50_ (Table 1). Thus, measuring viral DNA in batch’s supernatants is insufficient to correctly assign the infectivity of a batch and therefore biological assays should be performed to accurately determine viral titers.

The average intra-assay coefficient of variation (CV) for Q-PCR TCID_50_ was 9%, determined by parallel duplicate extractions and Q-PCRs of three TCID_50_ culture plates. For ocular TCID_50_ the intra-assay CV was 45%, determined by a total of twelve TCID_50_ culture plates read by two independent assessors. The intra-assay CV was 14% for IFA TCID_50_ determined by two parallel staining of cells from two runs. For the infectious units approach the intra-assay CV was 43% determined by four parallel staining of cells from one run. The average inter-assay CV was 73% for Q-PCR TCID_50_ and 66% for ocular TCID_50_, determined for three virus batches run five, three and three times respectively. For IFA TCID_50_ the inter-assay CV was 25%, determined for one batch run three times. For the infectious units approach the inter-assay CV was 77%, determined by three separate runs for one batch.

In summary, the Q-PCR TCID_50_ method described here correlates well with expression of viral proteins and thus has high specificity for infectious dose. It is more robust than ocular TCID_50_, IFA TCID_50_ and the infectious units approach, based on the intra-assay CV values. The intra-assay CV is in this setting a measure of how precise a certain read-out approach is and therefore is the most accurate value for comparisons of the different methods. To adapt the method, the cut point needs to be determined for every viral strain tested and every cell line used. The Q-PCR read-out approach is more laborious than ocular inspection, but in our opinion considerably less laborious than IFA TCID_50_ and the infectious units approach. It is more expensive in terms of laboratory resources than ocular inspection, IFA TCID_50_ and the infectious units approach. However, our data stresses the importance of performing biological assays to accurately determine viral titers, which might warrant the cost and labor. Furthermore, better standardization of viral titer assessment methods used within the HHV-6 field might increase the concordance between different studies.

## Abbreviations

HHV-6: Human herpesvirus 6; TCID_50_: 50% tissue culture infectivity dose; CPE: Cytopathic effects; IFA: Immunofluorescence assay; Q-PCR: Real-time quantitative PCR; CV: Coefficient of variation; SEM: Standard error of the mean, MOI, multiplicity of infection.

## Competing interests

The authors declare that they have no competing interests.

## Authors' contributions

RG, EE and AFH designed the experiments, RG and EE analyzed the results and performed the experiments, RG wrote the manuscript, EE and AFH contributed in critical discussions of the results and writing of the manuscript, AFH supervised the study, all authors read and approved the final manuscript.

## Supplementary Material

Additional file 1 **Figure S1.** Ocular inspection of HHV-6A (GS strain) infected HSB-2 cells. Dilutions and positive (+) or negative (−) results in Q-PCR TCID_50_ assessments are indicated. Undil: undiluted virus supernatant, 1:5; five times dilution of the virus supernatant, 1:5^2; 25 times dilution of the virus supernatant dilution etcetera. The figure shows one representative culture of sextuplicates for every dilution and of twelve runs of TCID_50_ assessment by ocular inspection for enlarged cells using phase contrast microscopy, 10 times enlargement. Click here for file
