# Supplementary figures and images for: Development and validation of a Q-PCR based TCID50 method for human herpesvirus 6
Source: Virol J. 2012 Dec 18;9:311. doi: 10.1186/1743-422X-9-311 (PMC3546908; doi:10.1186/1743-422X-9-311)

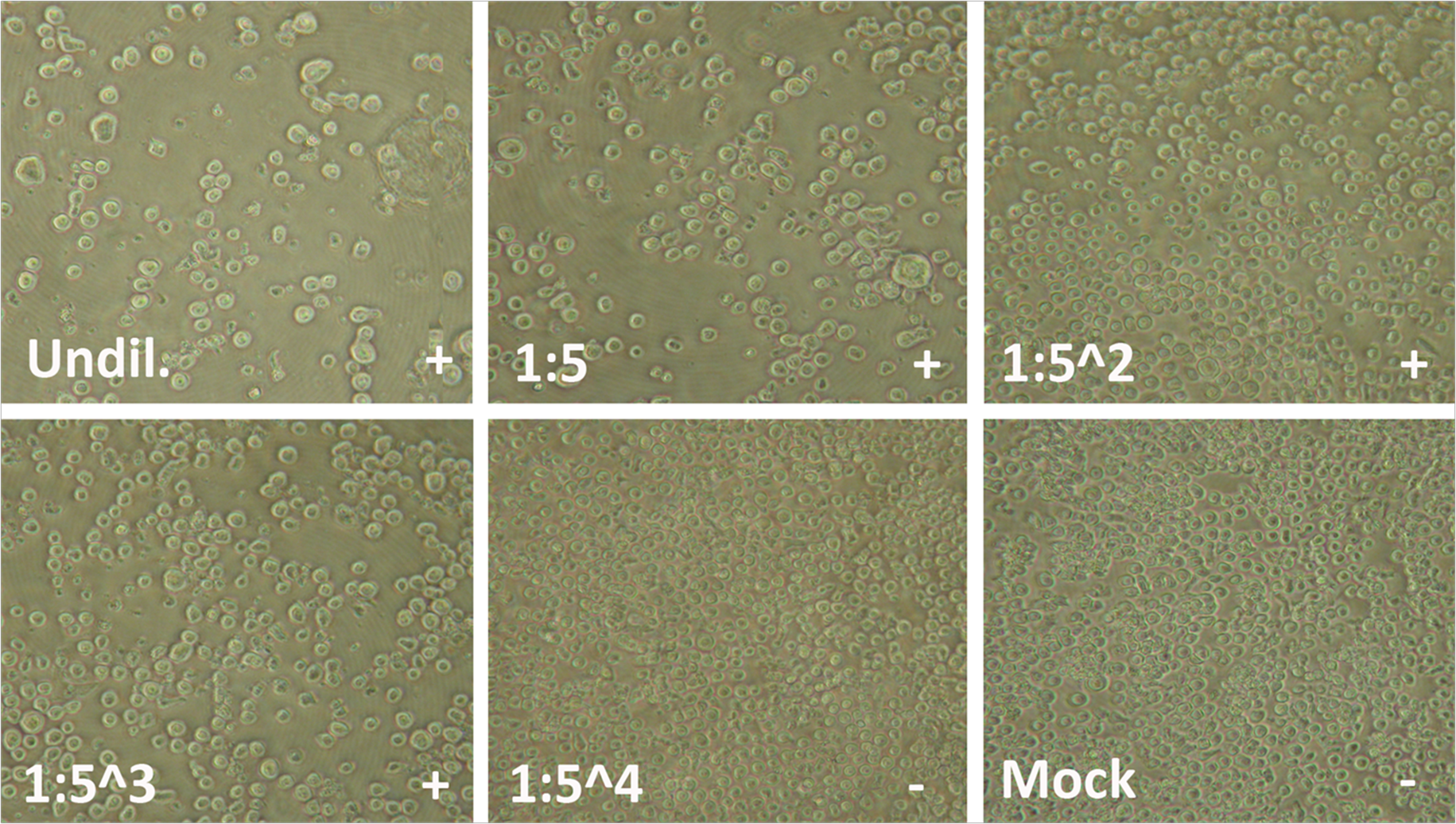

Supplement: Additional file 1 — Figure S1. Ocular inspection of HHV-6A (GS strain) infected HSB-2 cells. Dilutions and positive (+) or negative (−) results in Q-PCR TCID50 assessments are indicated. Undil: undiluted virus supernatant, 1:5; five times dilution of the virus supernatant, 1:5^2; 25 times dilution of the virus supernatant dilution etcetera. The figure shows one representative culture of sextuplicates for every dilution and of twelve runs of TCID50 assessment by ocular inspection for enlarged cells using phase contrast microscopy, 10 times enlargement. [file 1743-422X-9-311-S1.tiff]
